# Evaluating the effect of ethnicity and amyloid on gait speed in the Healthy Aging Brain Study‐Health Disparities Study

**DOI:** 10.1002/alz70857_106776

**Published:** 2025-12-25

**Authors:** Alexandra M Reed, Andrew Hooyman, Edward Ofori, Sydney Y Schaefer

**Affiliations:** ^1^ Arizona State University, Tempe, AZ, USA; ^2^ Chapman University, Orange, CA, USA; ^3^ Arizona State University, Phoenix, AZ, USA

## Abstract

**Background:**

Many novel Alzheimer's disease (AD) biomarkers have been studied in non‐Hispanic White populations, despite Hispanic/Latino adults being 1.5 times more likely to develop AD and receiving a later diagnosis. Equitable, accessible assessments are needed to identify individuals at risk earlier. While cognitive screening monitors early AD signs, neuropsychological tests are often inequitable across ethnicity, race, and other social determinants of health. However, we have shown that performance‐based motor assessments may be less susceptible to demographic factors than memory tests, and may be more equitable for screening older adults. Gait speed has been linked to elevated amyloid in non‐Hispanic White adults, but this relationship in Hispanic/Latino adults is unknown. This study compared the relationship between gait speed and amyloid in cognitively unimpaired non‐Hispanic White and Hispanic/Latino older adults.

**Method:**

This study included 597 adults (67.39% female, mean age: 63.47±8.51, 46.66% Hispanic) with a Clinical Dementia Rating of 0 from Wave 1 of the Healthy Aging Brain Study‐Health Disparities. Multiple linear regression was used to analyze the effects of ethnicity, sex, age, diabetes, hypertension, and amyloid on gait time (time to walk 4 meters, where lower is better), with interactions between ethnicity and amyloid, and ethnicity and diabetes. The interaction between age and gender was also explored. Amyloid positivity was determined using the florbetaben global standardized uptake value ratio (SUVR) threshold (>1.08). Gait time was evaluated as a continuous variable (seconds), while sex (M/F), diabetes (Y/N), hypertension (Y/N), and amyloid status (positive/negative) were categorical variables.

**Result:**

Non‐Hispanic White adults were older than Hispanic/Latino adults (mean age: 66.94±.7.92 vs 59.49±.7.37, *p* < .05), yet had significantly lower gait times (mean gait times: 3.87±0.83 sec vs 4.11±0.85 sec, *p* = .0028). Differences in gait time due to ethnicity was not explained by diabetes (*p* = .32), hypertension (*p* = .87), amyloid (*p* = .80), or an ethnicity by amyloid interaction (*p* = .87). Although the model was significant (*p* < .0001), little variance was explained (adj *R* ^2^ = .07).

**Conclusion:**

Gait speed may reflect risk factors associated with AD and related dementias, but it was not associated with AD‐specific pathology. Future research will explore gait time's susceptibility to social determinants of health.

